# Comparative Analysis of Whole-Transcriptome RNA Expression in MDCK Cells Infected With the H3N2 and H5N1 Canine Influenza Viruses

**DOI:** 10.3389/fcimb.2019.00076

**Published:** 2019-03-26

**Authors:** Pan Tao, Zhangyong Ning, Xiangqi Hao, Xi Lin, Qingxu Zheng, Shoujun Li

**Affiliations:** ^1^College of Veterinary Medicine, South China Agricultural University, Guangzhou, China; ^2^Guangdong Provincial Key Laboratory of Prevention and Control for Severe Clinical Animal Diseases, Guangzhou, China; ^3^Guangdong Technological Engineering Research Center for Pet, Guangzhou, China

**Keywords:** canine influenza, H3N2, H5N1, deep sequencing, total transcriptome

## Abstract

This study aimed to detect changes in the complete transcriptome of MDCK cells after infection with the H5N1 and H3N2 canine influenza viruses using high-throughput sequencing, search for differentially expressed RNAs in the transcriptome of MDCK cells infected with H5N1 and H3N2 using comparative analysis, and explain the differences in the pathogenicity of H5N1 and H3N2 at the transcriptome level. Based on the results of our comparative analysis, significantly different levels of expression were found for 2,464 mRNAs, 16 miRNAs, 181 lncRNAs, and 262 circRNAs in the H3N2 infection group and 448 mRNAs, 12 miRNAs, 77 lncRNAs, and 189 circRNAs in the H5N1 infection group. Potential functions were predicted by performing Gene Ontology (GO) and Kyoto Encyclopedia of Genes and Genomes (KEGG) analyses of the target genes of miRNAs, lncRNAs and circRNAs, and the ncRNA-mRNA regulatory network was constructed based on differentially expressed RNAs. A greater number of pathways regulating immune metabolism were altered in the H3N2 infection group than in the H5N1 infection group, which may be one reason why the H3N2 virus is less pathogenic than is the H5N1 virus. This study provides detailed data on the production of ncRNAs during infection of MDCK cells by the canine influenza viruses H3N2 and H5N1, analyzed differences in the total transcriptomes between H3N2- and H5N1-infected MDCK cells, and explained these differences with regard to the pathogenicity of H3N2 and H5N1 at the transcriptional level.

## Introduction

Influenza viruses belong to the *Orthomyxoviridae* family of RNA viruses that cause influenza in humans or animals, and the viral genome is composed of a single negative strand of segmented RNA. To date, four types of influenza viruses have been identified: influenza A virus (IAV), influenza B virus (IBV), influenza C virus (ICV), and most recently influenza D virus (IDV) (Hause et al., 2013, 2014).

IAV, which causes acute respiratory diseases in many hosts such as birds, humans and pigs, is an important pathogenic microorganism worldwide. Moreover, IAV has a wide host specificity and infects a variety of hosts. Influenza has been divided into avian influenza, swine influenza, human influenza and canine influenza. In the early days, IAVs were believed to be unable to infect dogs under natural conditions, but several subtypes were subsequently isolated from dogs with respiratory symptoms. Currently, two main subtypes of canine influenza virus (CIV) have been identified: equine-origin H3N8 (Crawford et al., 2005; Daly et al., 2008; Kruth et al., 2008; Kirkland et al., 2010) and avian-origin H3N2 (Song et al., 2008; Lee et al., 2009; Li et al., 2010). In addition, various subtypes of IAVs are reported to be able to infect dogs, including influenza A H1N1 pdm09 viruses, H5N1 avian influenza viruses (AIVs), H5N2 subtypes of AIVs, reassortants of wild-type H3N1 IAVs and H9N2 subtypes of AIVs (Songserm et al., 2006; Dundon et al., 2010; Lin et al., 2011; Guang-jian et al., 2012; Song et al., 2012; Sun et al., 2013).

Avian-origin H3N2 CIV, which was initially circulating only in Asian countries (Song et al., 2009; Li et al., 2010; Li G. et al., 2018), has now spread to the United States and the rest of the world (Pulit-Penaloza et al., 2017; Voorhees et al., 2017). H3N2 CIV causes sneezing and clinical symptoms of typical respiratory diseases, such as runny nose, cough and fever, as well as damage to many other organs outside the respiratory tract, in dogs (Luo et al., 2018; Zheng et al., 2018).

The highly pathogenic avian influenza (HPAI) H5N1 virus was first reported in Thailand in October 2004 in dogs with severe pulmonary congestion and edema and a bloody nose (Songserm et al., 2006). An epidemiological survey of 629 village dogs in Thailand found that approximately one-quarter had antibodies against H5N1, indicating that they were infected with the virus or had been infected in the past (Butler, 2006). Our laboratory also isolated an avian influenza H5N1 virus from a dog in 2013. The dogs that were infected with the highly pathogenic AIV H5N1 subtype showed anorexia, dyspnea, cough, conjunctivitis and a brief increase in body temperature within 2 days, but the virus did not spread between dogs (Maas et al., 2007; Giese et al., 2008). The highest viral replication titer of the nose swab was 6.3 log_10_TCID_50_/mL on average, and lung lesions in the H5N1 infection group were more severe than those in the H3N2 infection group (Zheng et al., 2018).

Why is the H5N1 influenza virus more pathogenic than H3N2, and why does it lead to a more severe inflammatory response? Current studies on H3N2 and H5N1 avian-origin CIV transcriptomes are limited to miRNAs and mRNAs (Fu et al., 2018; Zheng et al., 2018), whereas no studies have examined changes in mRNAs, miRNAs, lncRNAs, and circRNAs or performed a detailed analysis of the correlations between ncRNA and mRNA levels. In the present study, we analyzed the entire transcriptome to obtain an understanding of the mechanism of pathogenicity and differences in inflammation between the viruses and to provide guidance for future treatment.

Dogs are one of the most numerous domesticated animals and often serve as companion animals to humans. Because dogs can be infected with both avian and human influenza viruses, they are likely to be a “mixing vessel” for genetic rearrangement of influenza viruses, making the study of canine influenza important for public and human health (Gonzalez et al., 2014; Zhu et al., 2015).

## Materials and Methods

### Viruses and Cells

The H3N2 (A/canine/Guangdong/B/2013) and H5N1 (A/canine/Guangdong/01/2013) CIVs were isolated in 2013 from dogs with severe respiratory symptoms in Guangdong, China, and preserved in our laboratory. Madin-Darby canine kidney (MDCK) cells were obtained from American Type Culture Collection (ATCC) and propagated in Dulbecco's Modified Eagle's Medium (DMEM) supplemented with 10% fetal bovine serum (FBS) at 37°C in a 5% CO_2_ atmosphere. Viruses were propagated in the MDCK cells at 37°C and 5% CO_2_ for 48 h. All experiments with live viruses were performed in an enhanced animal biosafety level 3 facility at the South China Agricultural University. The protocol number was SYXK (YUE) 2016-0136.

### Sample Collection and RNA Isolation

MDCK cells were cultured with DMEM containing 10% FBS in a 37°C incubator with a 5% CO_2_ atmosphere. Upon reaching 90% confluence, MDCK cells were infected with the H3N2 (MOI = 0.1) and H5N1 (MOI = 0.1) influenza viruses, and the viruses used in these infections were purified using a sucrose gradient. Then, the cells were cultured with DMEM containing 2% FBS in a 37°C incubator with a 5% CO_2_ atmosphere for 24 h before being harvested. The control group was cultured without virus under the same conditions (independent triplicate experiments). Total RNA was isolated from MDCK cells using TRIzol (Takara) according to the manufacturer's protocol. RNA concentrations were detected using a Qubit2.0 fluorometer (Invitrogen), and the RNA integrity and genomic DNA contamination were detected by separating the samples on an agarose gel. RNA concentrations and purity were determined by measuring the OD, A260/A280 (>1.8) and A260/A230 (>1.6). RNA samples were stored at −80°C until further use.

### RNA Sequencing and Data Analysis

Small RNA library construction: T4 RNA ligase 2 (New England Biolabs) was used to connect the 3′-end connector to the RNA. Reverse transcription primer hybridization: T4 RNA ligase 1 (New England Biolabs) was used to connect the 5'-end connector to the RNA. Reverse transcription reaction: The final library product was obtained by PCR amplification of the reverse transcription product. Construction of a chain-specific library for ribosome removal: Sequencing libraries were generated using RNase R digestion and rRNA-depleted RNAs. The library preparations were sequenced on an Illumina Hiseq™ platform (repeated 3 times). The raw sequencing data were analyzed with FastQC using cutadapt to remove joints and trimmomatic to remove low-quality bases and reads at both ends.

### Screen of Differentially Expressed mRNAs, miRNAs, lncRNAs, and circRNAs and Clustering Analysis

The differences in expression among mRNAs, lncRNAs and circRNAs were analyzed using DESeq2, and the differential expression of miRNA was analyzed using edgeR (Anders and Huber, 2010). The results of the differential analyses were visualized, with a *p* < 0.05 and multiple differences >2 as screening conditions. A Venn diagram and a heat map were constructed, and a clustering analysis was performed based on the results of the differential expression analysis. A network diagram was drawn based on the correlations between miRNA and mRNA, lncRNA and mRNA, and circRNA and mRNA expression.

### Annotation of GO Terms and Analysis of KEGG Signaling Pathways

The differentially expressed miRNA, lncRNA, and circRNA target genes and mRNAs were annotated using GO and analyzed for enriched KEGG signaling pathways.

### Analysis of the ceRNA Network (ceRNET)

The circRNA-miRNA-mRNA ceRNET and the lncRNA-miRNA-mRNA ceRNET were constructed after determining the negative regulatory relationship between the differentially expressed miRNAs and their differentially expressed target genes (mRNAs/lncRNAs and mRNAs/circRNAs).

### Real-Time qPCR

Significant regulatory pathways were selected from the ceRNET for RT-qPCR verification of differential expression. The cDNA templates were synthesized using PrimeScript^TM^ RT Master Mix (Perfect Real Time) (Takara, Otsu, Japan, Product no: RR036A). RT-qPCR was performed using the SYBR Premix Ex Taq^TM^ (Tli RNaseH Plus) (Takara, Otsu, Japan, Product no: TRR820A) and an LC480 Real-Time PCR System (Roche, Basel, Switzerland) in accordance with the manufacturer's specifications. Small RNA samples were isolated using the E.Z.N.A.^TM^ miRNA Kit (OMEGA BIO-TEK. Product no: R6842-01). The miRcute Plus miRNA First Chain cDNA Synthesis Kit (Tiangen, Beijing, China. Product no: KP211) was used for cDNA synthesis. RT-qPCR was performed using the miRcute Plus miRNA qPCR Detection Kit (Tiangen, Beijing, China, Product no: FP411) and an LC480 instrument (Roche, Basel, Switzerland). GAPDH was used as the endogenous control for mRNAs and lncRNAs, and U6 was used as the endogenous control for miRNAs. We used the 2^−ΔΔ*Ct*^ method to analyze the data. All samples were analyzed in triplicate, and the data are presented as means ± standard deviations (*n* = 3).

## Results

### Replication Kinetics of H3N2 and H5N1 Strains in MDCK Cells

**Figure 1:**
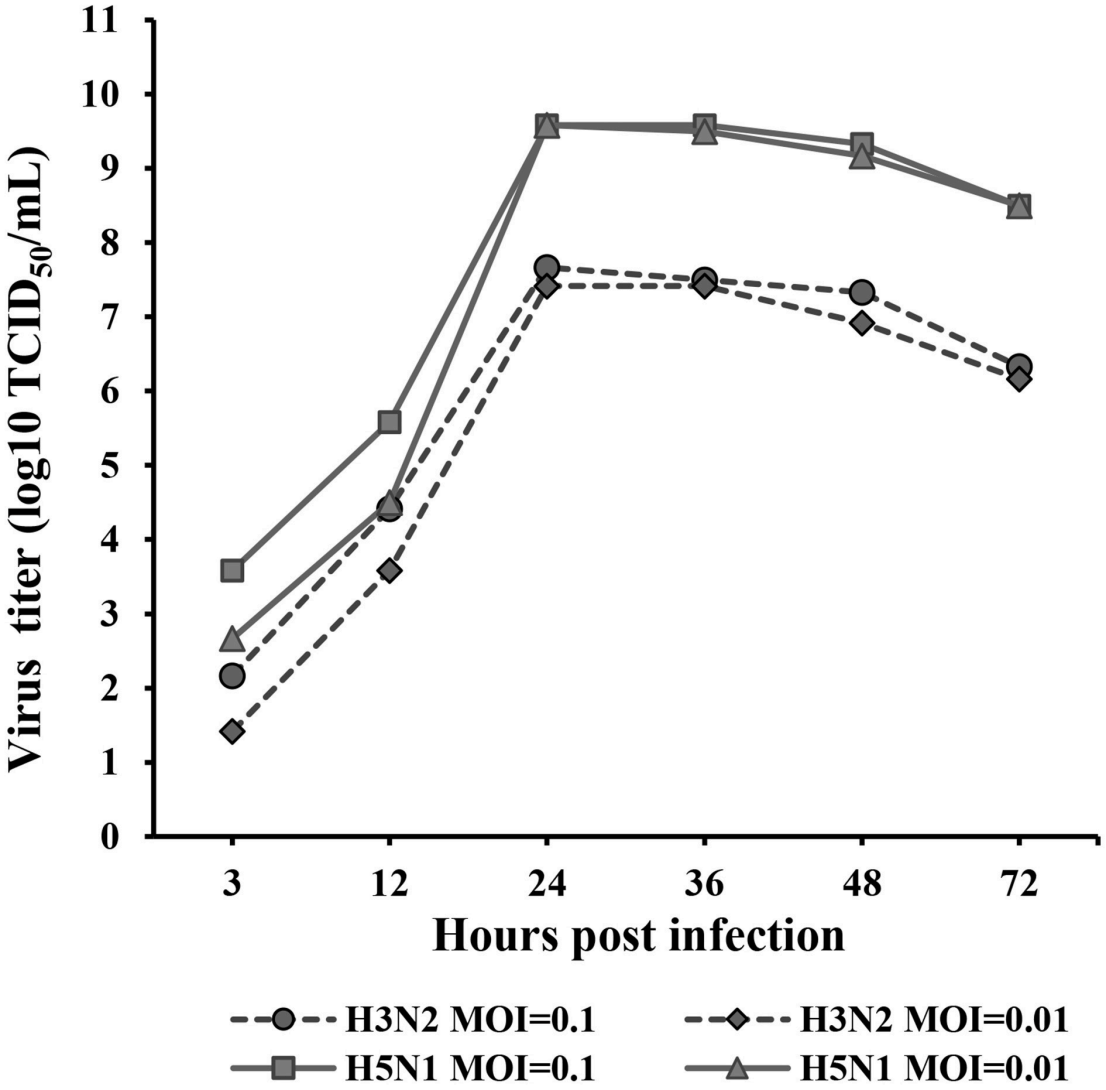
Replication kinetics of H3N2 and H5N1 in MDCK cells. Monolayers of MDCK cells were infected with each virus at MOIs of 0.1 and 0.01. Virus titers were determined using the TCID_50_ assay at 3, 12, 24, 36, 48, and 72 hpi. TPCK-trypsin (0.25 mg/mL) was added to the medium to promote the propagation of the H3N2 influenza A virus. The data were analyzed using one-way ANOVA (*p* < 0.05). Data are presented as the means ± SD of independent triplicate experiments.

The kinetics of H3N2 and H5N1 CIV replication were measured and analyzed based on the TCID_50_ at 3, 12, 24, 36, 48, and 72 hpi (Figure 1) in Reed-Muench. H3N2 and H5N1 CIVs replicated well in MDCK cells. H5N1 replicated significantly more vigorously than H3N2 (*p* < 0.01) at every assessed time point throughout the replication kinetics experiment. Viral replication was distinctly dose dependent for both H3N2 and H5N1 CIVs during the first 24 hpi, whereas the dose dependency was negligible from 24 to 72 hpi. The peak titers of the H5N1 CIV reached up to 10^9.6^ of the TCID_50_/mL at 24 hpi, whereas H3N2 CIV replicated significantly less rapidly, with peak virus titers reaching only 10^7.7^ of the TCID_50_/mL.

### Analysis of Differentially Expressed mRNAs, miRNA, lncRNAs, and circRNAs

**Figure 2:**
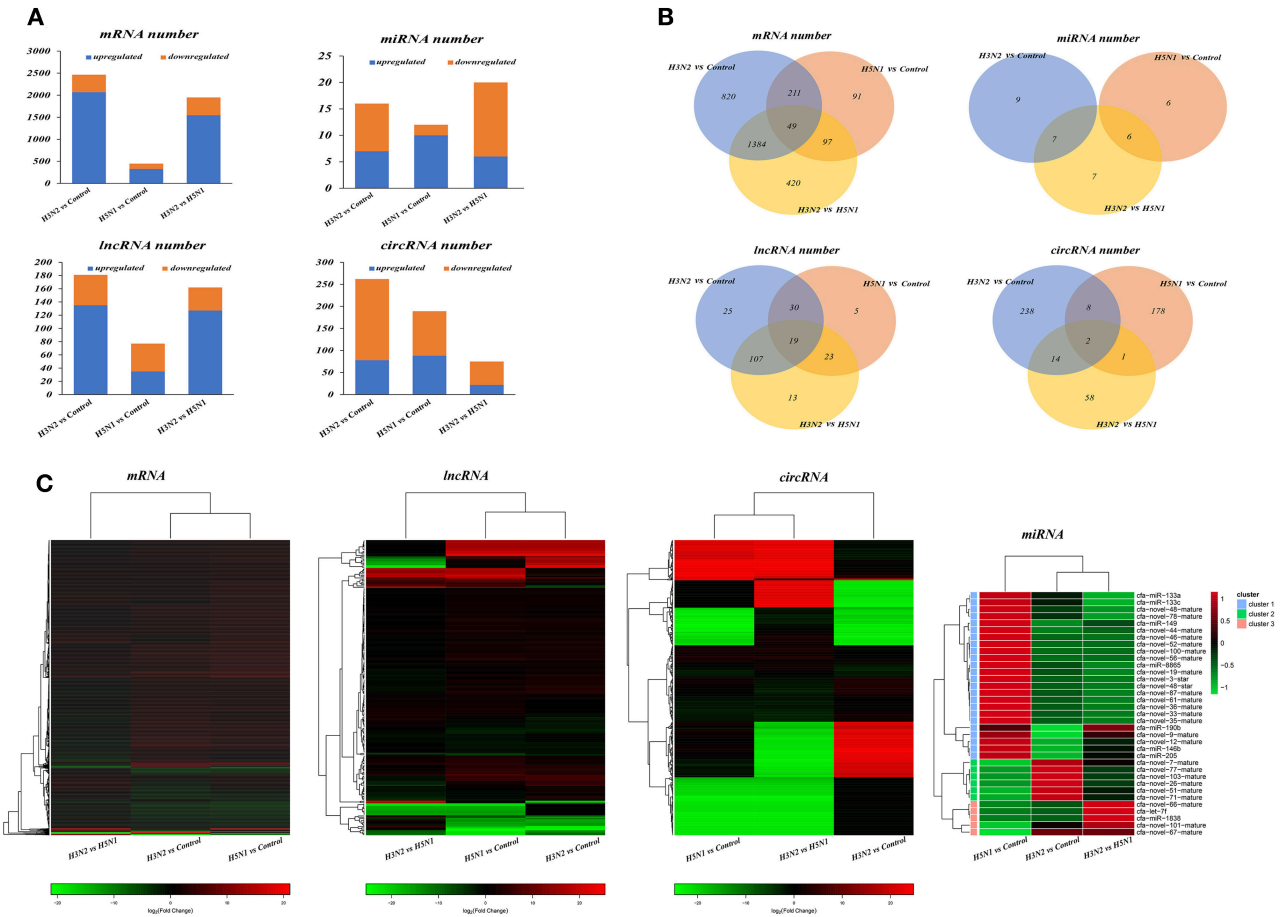
**(A)** Statistical histogram of the differential expression analysis. The horizontal axis represents the comparison and the vertical axis shows the number of differentially expressed transcripts. Orange represents downregulated transcripts, and blue represents upregulated transcripts. **(B)** Differentially expressed transcripts. In the Venn diagram, different comparison groups are represented by different colors. The numbers in the figure represent specific or common differences in the number of transcripts. The overlapping areas represent the number of differentially expressed transcripts shared by different comparison groups; the non-overlapping areas represent the number of differentially expressed transcripts that were unique to the different comparison groups. **(C)** Heat map of differentially expressed transcripts based on fold changes. Each row represents a transcript and each column represents a comparison group. Red indicates upregulated expression, and green indicates downregulated expression.

Through comparative analysis, we found that 2,464 mRNAs, 16 miRNAs, 181 lncRNAs, and 262 circRNAs were significantly differentially expressed in the H3N2 group compared with the control group and that 448 mRNAs, 12 miRNAs, 77 lncRNAs and 189 circRNAs were differentially expressed in the H5N1 group compared with the control group. Moreover, 1,950 mRNAs, 20 miRNAs, 162 lncRNAs, and 75 circRNAs were differentially expressed in the H3N2 group compared with the H5N1 group (Table 1, Figure 2 and Supplementary Material).

**Table 1:** Differential expression profiles of mRNAs, miRNAs, lncRNAs, and circRNAs.

**Group**	**Type of RNA**	**Up**	**Down**	**Total**
H3N2 vs. Control	mRNA	2,068	396	2,464
	miRNA	7	9	16
	lncRNA	135	46	181
	circRNA	78	184	262
H5N1 vs. Control	mRNA	328	120	448
	miRNA	10	2	12
	lncRNA	35	42	77
	circRNA	88	101	189
H3N2 vs. H5N1	mRNA	1,546	404	1,950
	miRNA	6	14	20
	lncRNA	127	35	162
	circRNA	22	53	75

### Correlation Analysis of mRNAs and ncRNAs

**Figure 3:**
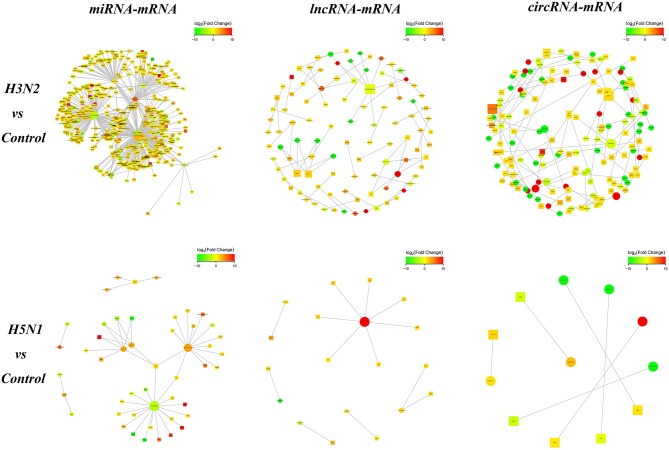
The network diagram of ncRNA and mRNA interactions. Square nodes indicate ncRNAs, round nodes indicate mRNAs, and the edges indicate the interactions between the two genes. The size of the node is proportional to the connectivity (degree) of the node; namely, the more edges are connected to the node, the larger the degree of the node, indicating that the gene is more important in the network. The color of the node represents the difference in gene expression in this group of samples, namely, the logFC value; green represents downregulation, red represents upregulation, and the color depth represents differences in the altered expression.

We analyzed the targeting relationship between differentially expressed ncRNAs and mRNAs to further study the correlation between the ncRNA-mRNA regulatory network in MDCK cells infected with CIVs. The analysis identified 706 differentially expressed miRNA-mRNA, 66 lncRNA-mRNA and 86 circRNA-mRNA pairs in the H3N2 group compared with the control group and 50 differentially expressed miRNA-mRNA, 13 lncRNA-mRNA, and 6 circRNA-mRNA pairs in the H5N1 group compared with the control group (Figure 3).

### Gene Ontology (GO) Functional Enrichment Analysis of Differentially Expressed Genes

**Figure 4:**
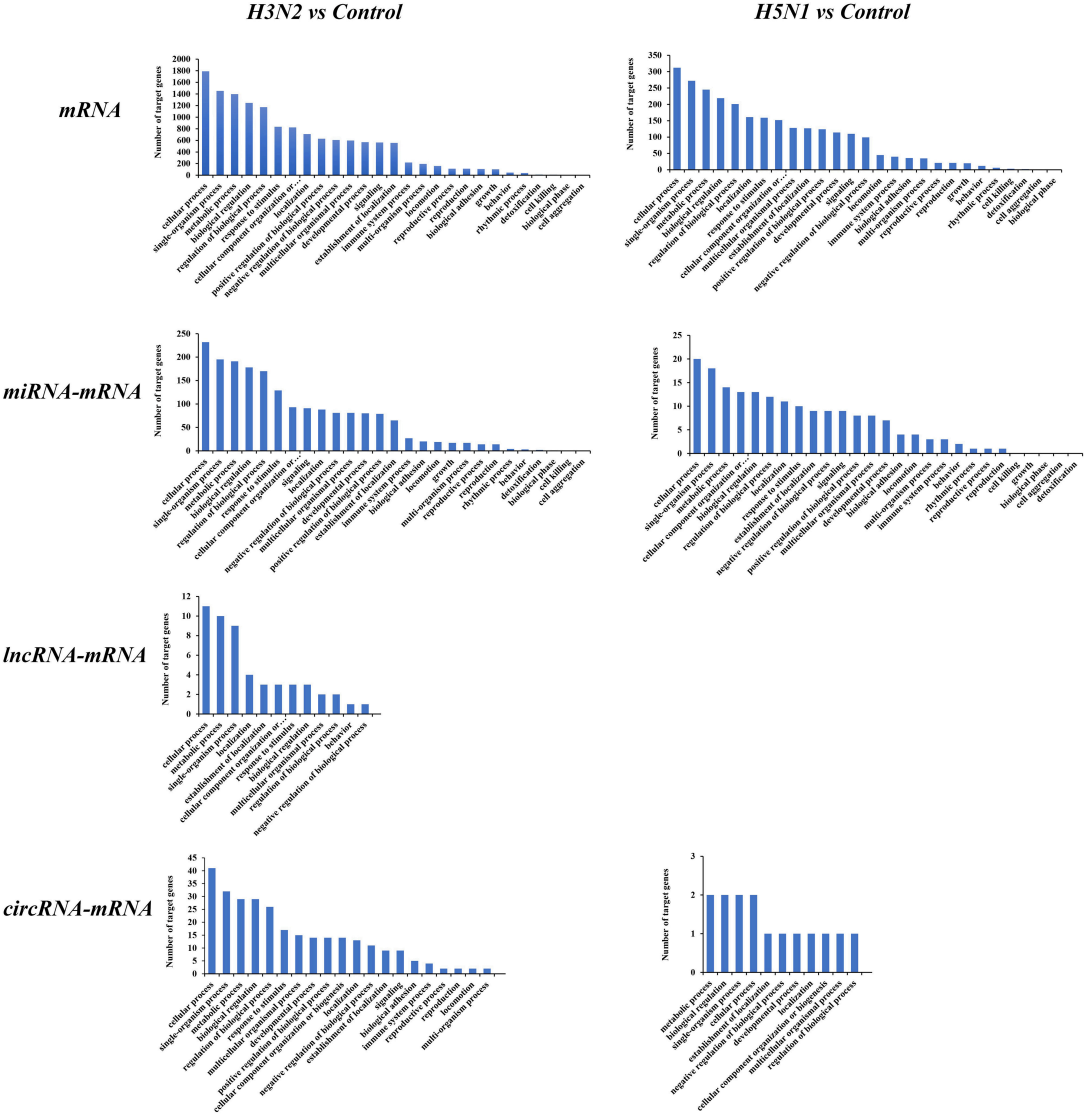
GO pathways of differentially expressed mRNAs and target genes of differentially expressed miRNAs, lncRNAs, and circRNAs. The horizontal axis shows the functional classification, and the vertical axis shows the number of genes in the classification.

We conducted a GO enrichment analysis and statistical analysis of biological processes to determine the functional classifications of these differentially expressed mRNAs and target genes of ncRNAs. A large number of genes in the H3N2 and H5N1 infection groups are involved in cellular process, single-organism process and metabolic process. However, many genes in biological processes were enriched in the H3N2 infection group, and each enriched functional classification also contained a large number genes. Based on the results of the GO enrichment analysis, miRNAs were more abundant than lncRNAs and circRNAs in terms of the number of target genes and functional classifications (Figure 4).

### Analysis of the Enrichment of Differentially Expressed Genes in Various Kyoto Encyclopedia of Genes and Genomes (KEGG) Pathways

**Figure 5:**
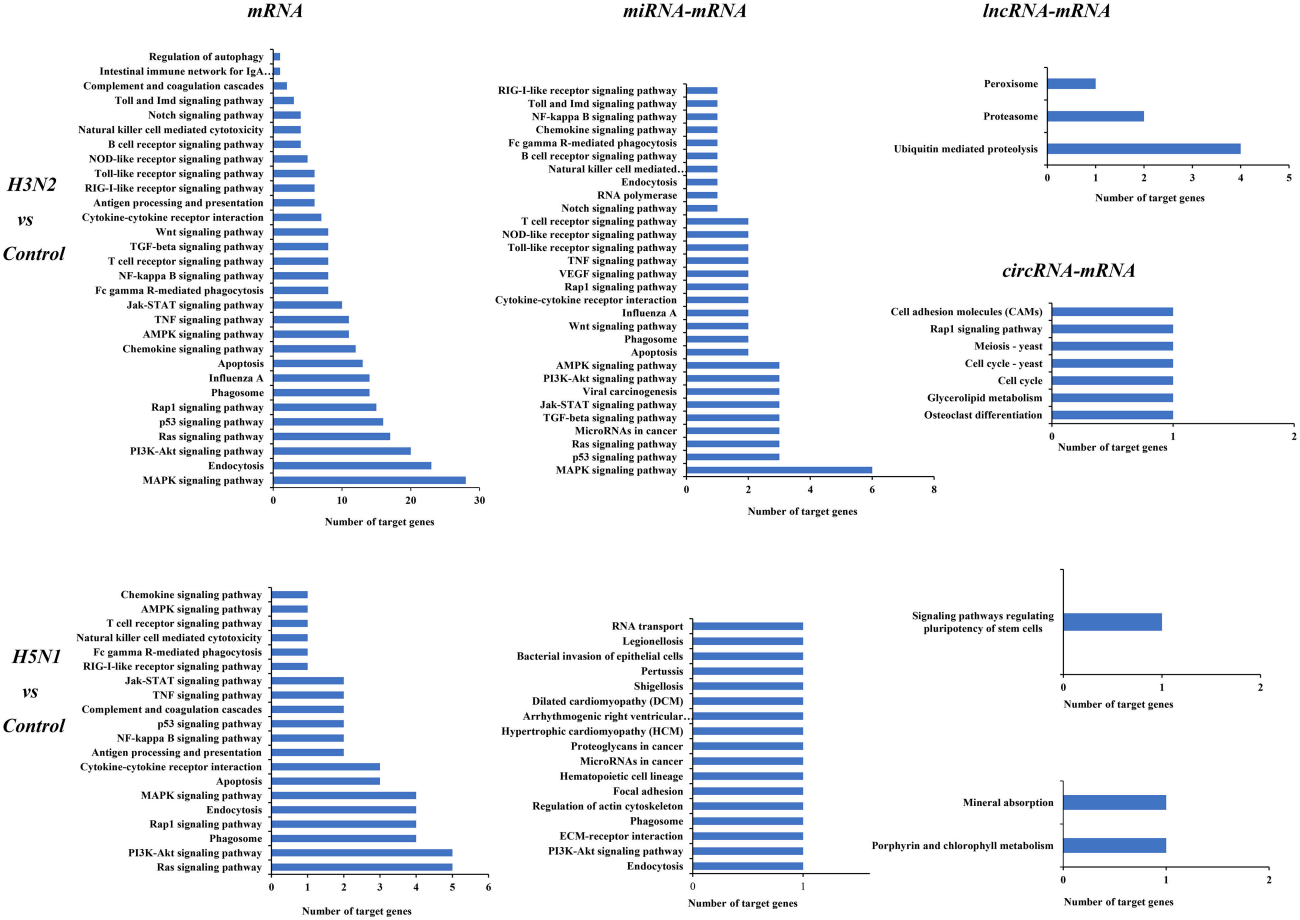
KEGG pathways of differentially expressed mRNAs and target genes of differentially expressed miRNAs, lncRNAs, and circRNAs. The horizontal axis shows the number of genes, and the vertical axis shows each pathway.

KEGG analysis was also performed for mRNAs and target genes of miRNAs, lncRNAs and circRNAs that were differentially expressed in cells infected with H3N2 and H5N1 to explore canine host resistance mechanisms to H3N2 and H5N1 infection and their differences. Then, we conducted a statistical analysis of cytokine and pathogen-host interaction pathways and found that a large number regulatory pathways and genes were enriched in the H3N2 infection group, particularly the MAPK signaling pathway, the endocytosis pathway, the p53 signaling pathway and other pathways with more obvious advantages. Notably, miRNAs can target more than one mRNA to regulate gene expression, indicating that miRNA-mediated targeted regulation of mRNA expression is more important than are the regulatory pathways involving lncRNAs and circRNAs (Figure 5).

### The Competing Endogenous RNA (ceRNA) Regulatory Network

**Figure 6:**
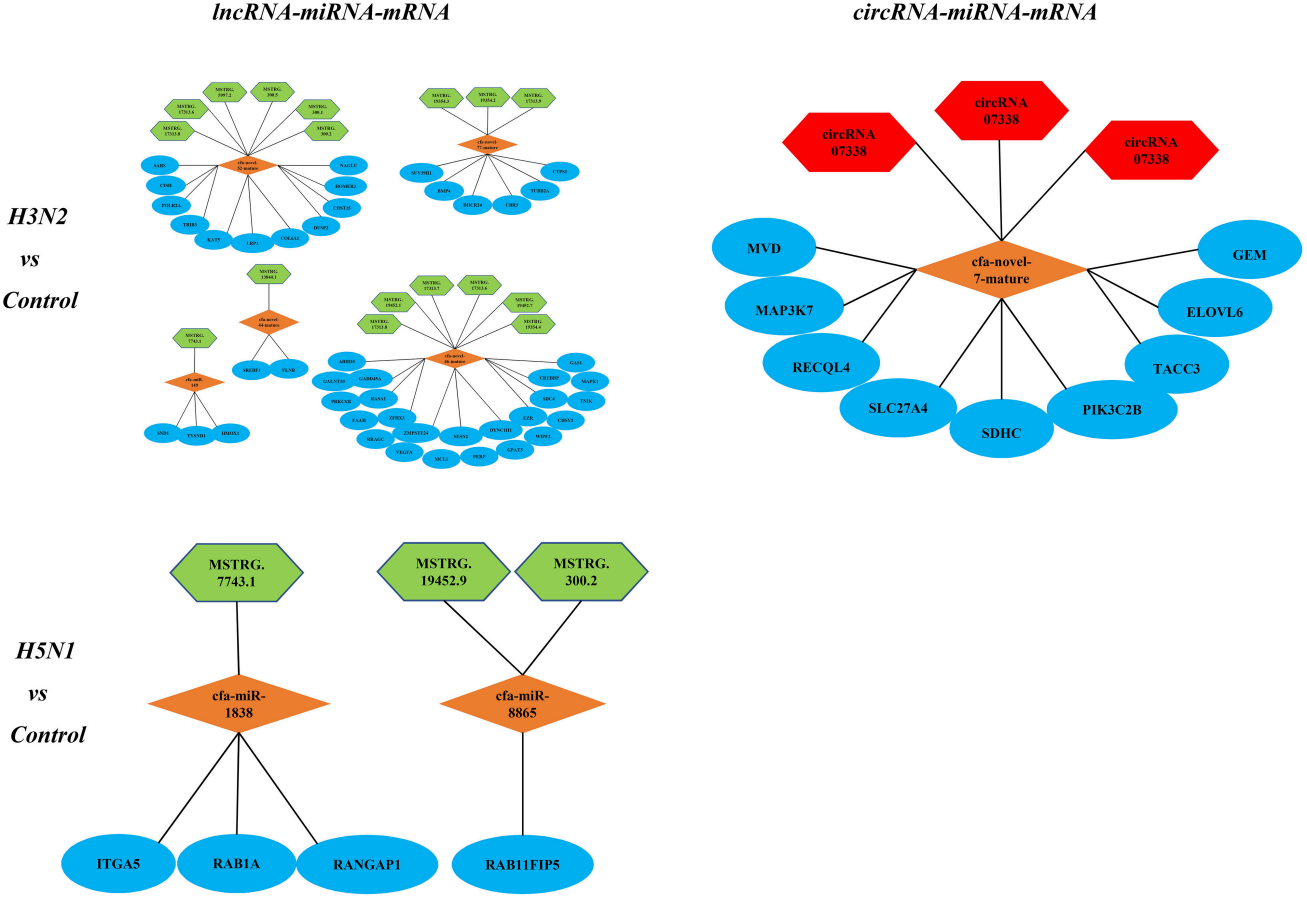
KEGG pathways associated with the lncRNA-miRNA-mRNA and circRNA-miRNA-mRNA regulatory networks; green represents lncRNAs, red represents circRNAs, orange represents miRNAs, and blue represents mRNAs.

Notably, lncRNAs and circRNAs limit miRNA-mediated regulation of target gene expression and act as miRNA sponges to indirectly regulate gene expression. According to the theory of ceRNAs, we constructed lncRNA-miRNA-mRNA and circRNA-miRNA-mRNA regulatory networks. In the comparison of the H3N2 and control groups, the lncRNA-miRNA-mRNA network contained 15 lncRNAs, 6 miRNAs and 237 mRNAs, and the circRNA-miRNA-mRNA network contained 3 circRNAs, 1 miRNA and 9 mRNAs. In the comparison of the H5N1 and control groups, the lncRNA-miRNA-mRNA network comprised 6 lncRNAs, 4 miRNAs and 18 mRNAs, and a circRNA-miRNA-mRNA regulatory network was not identified. Enrichment of mRNAs in the regulatory network was assessed via analysis of KEGG pathways (Figure 6).

### Differential Expression of ncRNAs and mRNAs Confirmed by RT-qPCR

**Figure 7:**
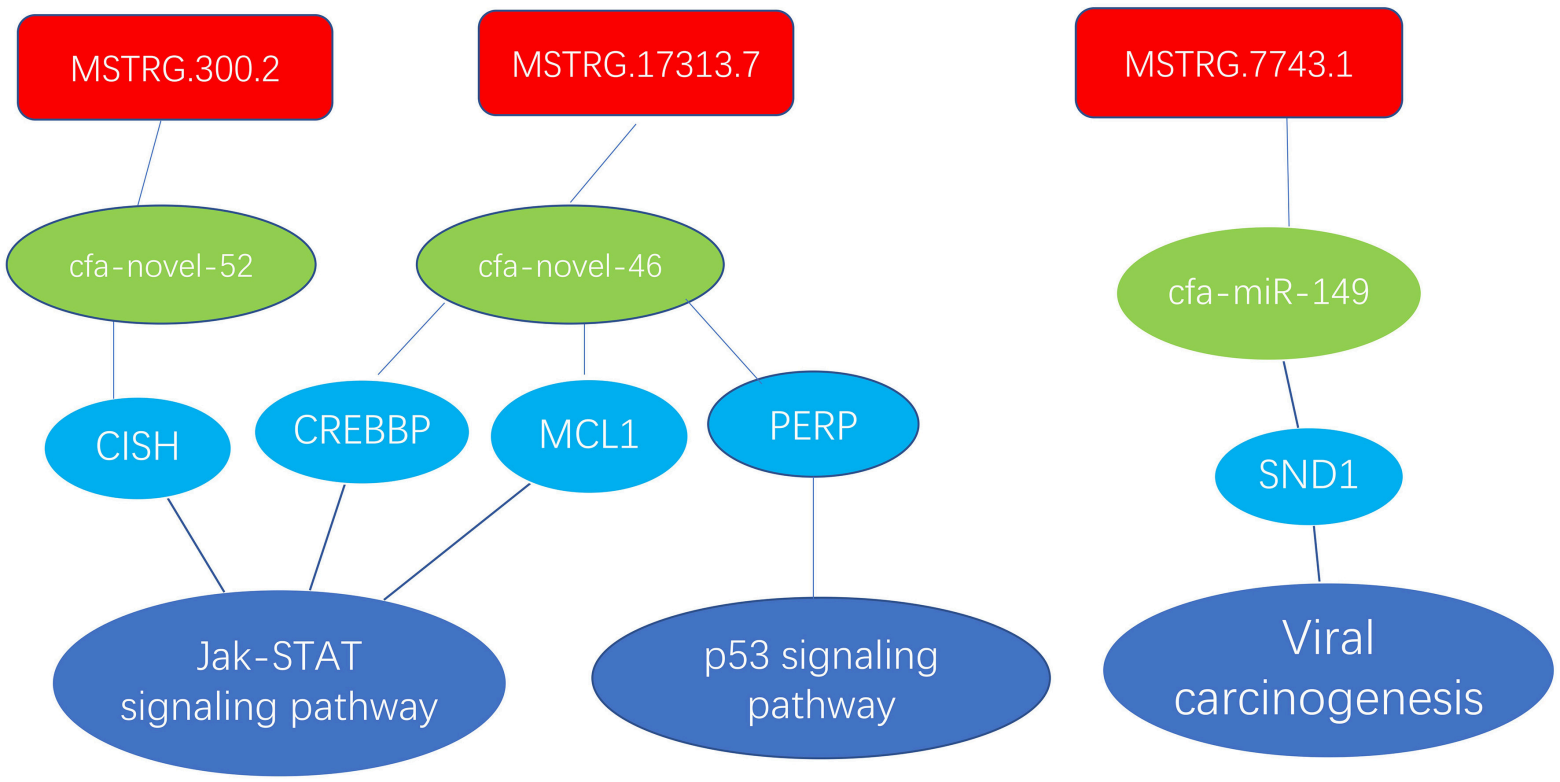
Enrichment of mRNAs in the Jak-STAT signaling pathway, p53 signaling pathway and viral carcinogenesis regulation network; lncRNAs are depicted in red, green represents miRNAs, and blue represents mRNAs.

In the ceRNA network, mRNAs in the Jak-STAT signaling pathway, p53 signaling pathway and viral carcinogenesis regulatory network were enriched and verified by RT-qPCR (primer sequences are shown in Table 2). The sequencing results were consistent with the trends in the qPCR verification results (Table 3 and Figure 7).

**Table 2:** Primer sequences used for real-time PCR.

**Gene name**	**Primer sequences (5^**′**^-3^**′**^)**
MSTRG.300.2	F: GATCCCGTGGGCGTTTACCCG
	R: GCAAGACACCCAACAGCGGCG
MSTRG.17313.7	F: GGAGTGCTGAGAAGACGGTCGAAC
	R: GCCGCGTTCTCCGTTAATGATCC
MSTRG.7743.1	F: AGGAGCATCTCGGGCTTTTCA
	R: CTTTACCGCTCCATCAACGCA
cfa-novel-52-mature	F: GCCCCCCGGGGGGGCGG
cfa-novel-46-mature	F: GCGGCGGCGGGGAGGGT
cfa-miR-149	F: AGACCGAGGCACAGAAGTGAGGG
U6	F: ACTAAAATTGGAACGATACAGAGA
CISH	F: TTCTTTGCTGGCTGTGGAGCG
	R: GCCTCACTGGCGGTAATGGAA
CREBBP	F: CTTTAAGCCAGAGGAGTTACGC
	R: GGATGTCTTGCGGTTATAGAGC
MCL1	F: ACTGGGGCAGGATTGTGACTCT
	R: GCCAGTCTCGTTTCGTCCTTAC
PERP	F: CCCGAGAGTTCCTTAGCACA
	R: ATGATGTCGAAGGCGATGGC
SND1	F: TAGAGGTGGAGGTAGAGAGCAT
	R: GACAGCAGGGATTTGTAGTAGG
GAPDH	F: AAATGGGGTGATGCTGGTGCT
	R: CATCAGCAGAAGGAGCAGAGA

**Table 3:** Relative RNA expression of selected differentially expressed genes (DEGs) determined using RNA-seq and quantitative real-time PCR analyses.

**lncRNA_name/miR_name/mRNA_name**	**Accession number**	**Illumina miRNA-seq (log_**2**_-fold change)**	**Regulation**	**Real-time PCR (log_**2**_-fold change)**
MSTRG.300.2		5.574059	Up	4.06^*^
MSTRG.17313.7		24.63398	Up	20.25^*^
MSTRG.7743.1		1.199492	Up	1.53^*^
cfa-novel-52-mature		−3.62139	Down	−4.52^*^
cfa-novel-46-mature		−2.7541	Down	−3.02^*^
cfa-miR-149		−1.71902	Down	−2.08^*^
Cytokine inducible SH2 containing protein (CISH)	ENSCAFT00000045397	1.291812	Up	1.06^*^
CREB binding protein (CREBBP)	ENSCAFT00000044601	1.619728	Up	2.05^*^
MCL1, BCL2 family apoptosis regulator (MCL1)	ENSCAFT00000019132	2.098362	Up	2.16^*^
PERP, TP53 apoptosis effector (PERP)	ENSCAFT00000043071	1.268355	Up	1.89^*^
Staphylococcal nuclease and tudor domain containing 1 (SND1)	ENSCAFT00000002687	1.176868	Up	1.39^*^

* *The statistical significance of differential gene expression with p < 0.05 (t-test)*.

## Discussion

In this study, a high-throughput sequencing technique was used to compare the entire transcriptomes of MDCK cells infected with the H5N1 and H3N2 viruses. Although fewer differentially expressed mRNAs were identified in the H5N1 infection group than in the H3N2 infection group, a higher proportion of mRNAs exhibited a more significant differential expression pattern in the H5N1 infection group. In the H5N1 group, 24 (log_2_-fold change > 10) of 448 mRNAs were differentially expressed mRNAs, while only 27 (log_2_-fold change > 10) of 2,464 mRNAs were differentially expressed in the H3N2 group, 10 of which were shared by the two groups. In the ncRNA-mRNA regulatory network, the H3N2 group had more regulatory pairs than did the H5N1 group. Thus, H3N2-infected MDCK cells may be able to resist the viral infection through more pathways to reach a balanced state. This finding also illustrates why H5N1 is more virulent than H3N2 at the RNA level.

In the circRNA-miRNA-mRNA regulatory network, we identified 3 circRNAs in the H3N2 infection group. One miRNA and 9 mRNAs had a targeted relationship, while the H5N1 infection group did not possess this regulatory network. In the lncRNA-miRNA-mRNA regulatory network, 15 lncRNAs, 6 miRNAs, and 237 mRNAs were identified in the H3N2 infection group. Six lncRNAs, 4 miRNAs, and 18 mRNAs were identified in the H5N1 infection group. Based on this result, H3N2 infection may induce broad regulation in MDCK cells, and the circRNA-miRNA-mRNA regulatory network is not common in influenza infection. Regarding GO and KEGG enrichment analyses, we also intuitively observed a greater number of GO annotations and enriched KEGG pathways in the H3N2 infection group than in the H5N1 infection group. In conclusion, we postulate that H5N1 is more virulent than H3N2 for several reasons. (1). H5N1 infects cells and causes intracellular cytokine storms, resulting in rapid cell death (Li X. et al., 2018). (2). Cells infected with H3N2 utilize more resistance pathways to eliminate the virus, while H5N1 infection activates relatively few pathways in cells, which showed the some differences in MDCK in antiviral responses between H3N2 and H5N1 at the RNA level of the transcriptome.

Notably, lncRNAs play important roles in many cellular activities, such as the regulation of epigenetics, the cell cycle and cell differentiation, and has become a hot topic in genetic research (Wapinski and Chang, 2011; Kwok and Tay, 2017). MiRNAs and their target genes have a variety of relationships (Perez et al., 2009; Fan and Wang, 2016; Nakamura et al., 2016), and misaligned miRNAs can be used as diagnostic and prognostic biomarkers (Okkenhaug and Vanhaesebroeck, 2003; Hale et al., 2010; Haneklaus et al., 2013). In the present study, we examined the lncRNA-miRNA-mRNA regulatory networks of the Jak-STAT signaling pathway, the p53 signaling pathway and the viral carcinogenesis pathway for RT-qPCR verification. The Jak-STAT signaling pathway, which is closely related to the type I interferon (IFN)-mediated innate immune response, is an important regulator of cell proliferation, differentiation, survival, motility, apoptosis, development and the immune response (Bartunek et al., 1999; Liu et al., 2010). Currently, p53 is the most widely analyzed functional transcription factor. The aggregation and induced activation of p53 are the core cell signaling events in a variety of stress-induced injury responses and have important regulatory roles in inhibiting cell cycle progression and inducing DNA damage repair, cell autophagy and apoptosis (Crighton et al., 2006; Duan et al., 2015; Sui et al., 2015). In the next step, we further explored the mechanisms of these pathways.

In this study, the total transcriptome of the MDCK cell model infected with CIVs was analyzed for the first time. The results further revealed the differences in pathogenicity between H3N2 and H5N1 at the RNA level of the transcriptome. However, this study has only analyzed the differences in the RNA levels at 24 hpi in canine cells and further studies are needed to analyze both earlier time points as well as the differences in the RNA transcriptome in dogs infected with the two types of influenza viruses.

## Author Contributions

PT, XH, XL, and QZ conducted the experiments. PT and ZN analyzed the data and wrote the paper. SL designed the experiments and revised the paper.

### Conflict of Interest Statement

The authors declare that the research was conducted in the absence of any commercial or financial relationships that could be construed as a potential conflict of interest.

## Supplementary Material

Supplementary Data Sheet 1 The original data.
